# 

**Published:** 1846-04

**Authors:** 


### CONTENTS

OF NO. IV.

### ORIGINAL COMMUNICATIONS.

### ESSAYS AND CASES.

Art. I.—Letters from Europe. By James C. Cross, M. D., late Professor of the Institutes of Medicine in Transylvania University. . . . . . . 253

Art. II.—An Essay on Diet in Health and Disease. By John Dawson, M.D., of Jamestown, Ohio. . 286

### REVIEWS.

Art. III.—Elements of Pathological Anatomy; Illustrated by colored Engravings and 250 Woodcuts. By Samuel D. Gross, M.D., Professor of Surgery in the Medical Institute of Louisville; Late Professor of Pathological Anatomy in the Medical Department of the Cincinnati College; Surgeon to the Louisville Marine Hospital, &c., &c. Second Edition, thoroughly revised and greatly enlarged. . . . . 315

### SELECTIONS FROM AMERICAN AND FOREIGN JOURNALS.

On the Acetate of Lead in Alvine Discharges. . . . 345

Statistics of the frequency of Cardiac and Pulmonary Complication in Acute Rheumatism. . . . . . 345

Case of Ileus. . . . . . . . . . . . . . 346

Syphilis. . . . . . . . . . . . . . . 347

Uterine Hemorrhage, . . . . . . . . . 247
Cases in Obstetric Practice, . . . . . . 352
Laudanum in the delirium occurring in Dothinenteritis. . 354
Report of the Committee of the New York State Medical Society concerning the National Medical Convention. 354
Animalcules in the Oil-tubes of the Skin. . . . 357
Warm moist Air in Bronchial Inflammation. . . . 358

ORIGINAL INTELLIGENCE.

Commencement in the Medical Institute. . . . 361
Analysis of the Boston Waters. . . . . . 362
Medical Obedience. . . . . . . . . 363
The Medical Profession in the United States. . . 364
Riddell on the Constitution of Matter. . . . . 365
National Medical Convention. . . . . . . 366
The Naturalist. . . . . . . . . . 366
Hypotheses in Medicine. . . . . . . 366
To Correspondents. . . . . . . . . 368
Books Received. . . . . . . . . 368

